# Prevalence of physical inactivity and barriers to physical activity among obese attendants at a community health-care center in Karachi, Pakistan

**DOI:** 10.1186/1756-0500-4-174

**Published:** 2011-06-06

**Authors:** Nafisa Samir, Sadia Mahmud, Ali Khan Khuwaja

**Affiliations:** 1 Department of Family Medicine and Community Medicine, Sultan Qaboos University Hospital, P.O. Box. 35, Al-Khoud 123 Muscat, Oman; 2 Department of Community Health Sciences, The Aga Khan University, Karachi, PO Box 3500, Stadium Road, Karachi 74800 Pakistan; 3 Department of Family Medicine, The Aga Khan University Hospital, PO Box 3500, Stadium Road, Karachi 74800 Pakistan

## Abstract

**Background:**

Overweight and obesity are significant public health problems worldwide with serious health consequences. With increasing urbanization and modernization there has been an increase in prevalence of obesity that is attributed to reduced levels of physical activity (PA). However, little is known about the prevalence of physical inactivity and factors that prohibit physical activity among Pakistani population. This cross-sectional study is aimed at estimating the prevalence of physical inactivity, and determining associated barriers in obese attendants accompanying patients coming to a Community Health Center in Karachi, Pakistan.

**Findings:**

PA was assessed by using international physical activity questionnaire (IPAQ). Barriers to PA were also assessed in inactive obese attendants. A pre-tested questionnaire was used to collect data from a total of 350 obese attendants. Among 350 study participants 254 (72.6%) were found to be physically inactive (95% CI: 68.0%, 77.2%). Multivariable logistic regression analysis indicated that age greater than 33 years, BMI greater than 33 kg/m^2 ^and family history of obesity were independently and significantly associated with physical inactivity. Moreover, there was a significant interaction between family structure and gender; females living in extended families were about twice more likely to be inactive, whereas males from extended families were six times more likely to be inactive relative to females from nuclear families. Lack of information, motivation and skills, spouse & family support, accessibility to places for physical activity, cost effective facilities and time were found to be important barriers to PA.

**Conclusions:**

Considering the public health implications of physical inactivity it is essential to promote PA in context of an individual's health and environment. Findings highlight considerable barriers to PA among obese individuals that need to be addressed during counseling sessions with physicians.

## Background

The prevalence of overweight and obesity has reached epidemic proportions [1] and has become a challenge for public health practitioners as well as for clinicians. According to World Health Organization (WHO) 1.5 billion adults are overweight, of these over 200 million men and 300 million women are obese worldwide [2]. Obesity is particularly high among South Asian population [3,4]. According to the National Health Survey of Pakistan (1990-1994), prevalence of overweight and obesity were estimated to be 25% and over 10% respectively [5]. This trend is constantly rising and recently, Khuwaja and Kadir in a community-based survey in Karachi reported that about half of the study participants were overweight and obese [6].

Overweight and obesity are the fifth leading risk for death globally; about 3.0 million adult deaths each year are attributed to these [2]. It is well documented that overweight is particularly associated with cardiovascular disease and its risk factors like hypertension, type 2 diabetes, dyslipidemia; metabolic and endocrinal diseases and musculoskeletal disorders [2,6,7]. Evidence also exists regarding association of obesity with various cancers [8]. However, these health risks, morbidities and mortality can be substantially prevented by maintaining optimal body weight [8,9].

Urbanization and modernization has mainly contributed to epidemic of obesity through reduced level of physical activity [1,10]. Despite the well-known benefits of regular physical activity, it is estimated that over 60% of the world's population is not physically active enough to gain health benefits [11]. Statistics for Pakistani population are similar; a study conducted in Pakistan found that majority of adults was physically inactive [6]. It is therefore imperative to identify the factors associated with physical inactivity, and the barriers to physical activity particularly among inactive obese individuals. Understanding these barriers may help in developing effective programs to meet public health recommendations for physical activity [12,13]. This study is aimed to assess the prevalence of physical inactivity among obese individuals, and to identify various socio-demographic factors and barriers associated with physical inactivity.

## Methods and material

This cross-sectional study was conducted from 1st February to 30th April 2007 at the Community Health Center (CHC) in the Aga Khan University Hospital (AKUH), Karachi. AKUH is a private sector teaching hospital. The majority of the physicians practicing in CHC are family physicians, and the preponderance of people who seek medical services belong to literate and middle-class economic communities residing in Karachi. Karachi is the largest city and the economic hub of Pakistan with an estimated population over 10 million people of diverse ethnic and socioeconomic groups [14].

The study population comprised of obese attendants accompanying patients coming to CHC at AKUH. In CHC nearly 250 patients get registered daily, we assumed that about 200 would be accompanied by attendants. In one day one data collector could interview only 10 attendants as the questionnaires were extensive and each interview took approximately 35 to 40 minutes. For each day of the data collection period the attendants in the waiting room were selected by systematic random sampling by three data collectors. After taking informal consent, height and weight of participating attendants were measured, and BMI (in kg/m^2^) was calculated by data collectors. Attendants who had BMI ≥ 25 kg/m^2 ^were assessed for other eligibility criteria. Participants with age between 18 & 69 years and BMI ≥ 25 kg/m^2 ^were included in the study. This age group was selected as IPAQ (instrument used in the study to assess physical activity) is specifically designed for individuals 18 to 69 years old [15]. The cutoff for BMI ≥ 25 kg/m^2 ^was used as this is the obesity cutoff defined by WHO for Asian population [16]. Pregnant women and those who were unable to give interview due to any disability were excluded. Those who met all eligibility criteria were invited to participate in the study, and their written informed consent was taken. The level of PA was measured using International Physical Activity Questionnaire (IPAQ) [15]. The obese attendants who were found to be inactive were further questioned regarding barriers to physical activity.

In 1998-99 IPAQ validation and reliability studies were conducted in 14 research centers in 12 countries on 6 continents using standardized methods and protocols. Overall, the IPAQ produced repeatable data, Spearman's correlation coefficient clustered around 0.8 [15]. IPAQ categorizes PA into three categories; low, moderate and high. In this study PA was considered as a binary variable; individuals with low levels of PA were considered as physically inactive, and those with moderate or high level were considered as physically active.

Sample size was calculated to estimate the proportion of physically inactive individuals among obese attendants using a 95% confidence interval with 5% error bound. We assumed that at least 70% of the obese were inactive [17]. The calculated sample size was 323 that was inflated to 350.

Data was collected through interviews of the study participants. One comprehensive pre-tested questionnaire was filled by data collectors for individual subjects. The questionnaire had two sections; the first section assessed socio-demographic characteristics and the level of PA using IPAQ and the 2^nd ^section comprised of barriers to PA, and was administered to only those obese attendants who were found to be physically inactive. IPAQ and the above mentioned questionnaire were translated from English into Urdu (local language) for the purpose of interviews. It was also back translated into English to assess if the original meaning was preserved.

Ethical approval for the study was obtained from the Institutional Ethical Review Committee (ERC, Aga Khan University). Informed consent was taken from study participants before collecting the data.

Information regarding the following barriers to PA among the obese inactive attendants was recorded. (These barriers were identified from previous studies [18]).

### Personal barriers

Lack of information, lack of motivation, lack of enjoyment, lack of skills and feeling embarrassed were measured on an ordinal scale. Physical illness was recorded as a binary variable (present or absent).

### Social barriers

They were also measured on an ordinal scale, and include lack of spouse's support, lack of family support, lack of children's support and lack of awareness.

### Environmental barriers

Lack of proper counseling, lack of accessibility to places for exercise, lack of cost effective facilities, lack of safe outdoor environment were measured on an ordinal scale, whereas lack of time was recorded as a binary variable (yes or no).

### Statistical Analysis

Epi Info and SPSS version 13 were used for data entry and data analysis respectively. Descriptive statistics were computed for socio-demographic characteristics. Proportion of physically inactive individuals among study participants was computed. Chi-square test of independence and T-test were conducted to assess association of PA status with categorical and continuous variables respectively. A final set of factors associated independently with physical inactivity, along with adjusted odds ratios, were obtained by multivariable logistic regression analysis. Frequencies of barriers to physical activity among inactive obese attendants were computed.

## Results

After a random selection 350 obese attendants were interviewed. Among 350 study participants 254 (72.6%) were found to be physically inactive (95% CI = 68.0%, 77.2%). Socio-demographic characteristics of the study participants are reported in Table 1. In Table 2 we report comparison of socio-demographic characteristics between the physically inactive and active group. Mean age and BMI in the physically inactive group were significantly higher than that in the physically active group. In the physically inactive group more subjects were currently married, lived in extended family structure and had a positive family history of obesity.

**Table 1:** Socio-demographic characteristics of study participants *(n = 350)*

Continuous Variables	Mean	S.D
Age	40.73	11.03
BMI	30.58	4.09
Income	Mean: 18872	18601.61
	Mode: 10000*	
	Median:13000*	

**Categorical variables**	**Frequency**	**Percentage**

***Gender***		
Male	163	46.6
Female	187	53.4
		
***Ethnicity***		
Urdu	135	38.6
Other ^‡^	215	61.4
		
***Marital status***		
Single	40	11.4
Married	302	86.3
Widowed	8	2.3
		
***Level of Education***		
Uneducated	47	13.4
Below Matric	77	22.0
Matric & Above	226	64.6
		
***Family structure***		
Nuclear ^†^	158	45.1
Extended	192	54.9
		
***Family history of obesity***		
Yes	222	63.4
No	128	36.6

* Mode & Median reported as the data was skewed. The unit of income is Rupees.

**^‡ ^**As people all over the country are coming to Karachi for better perspectives and opportunity many ethnic groups were found in the study which was merged together.

**^†^**Nuclear family system was defined as a household consisting of parents and their children; extended family system was defined as a household where multiple generations of a family were living together.

**Table 2:** Comparison of socio-demographic characteristics between physically inactive & physically active groups *(n = 350)*

Continuous variables	Physically inactive	Physically active	p - value
		
	Mean [SD]	Mean [SD]	
***Age***	41.74 [10.67]	38.05 [11.57]	0.01*
***BMI***	30.93 [4.35]	29.66 [3.15]	0.01*
***Income***	18495 [17413]	19871 [21499]	0.59^‡^
	Mode: 10000	Mode: 5000	
	Median: 15000	Median: 12000	

**Categorical variables**	**Physically inactive**	**Physically active**	**p - value**
		
	**Numbers [%]**	**Numbers [%]**	

***Gender***			
Male	121 [47.6]	42 [43.7]	0.52^†^
Female	133 [52.4]	54 [56.3]	
			
***Ethnicity***			
Urdu	93 [36.6]	42 [43.8]	0.22^†^
Other	161 [63.4]	54 [56.2]	
			
***Marital status***			
Single	20 [7.9]	20 [20.8]	<0.05^†^
Married	229 [90.1]	73 [76.1]	
Widow	5 [2.0]	3 [3.1]	
			
***Level of Education***			
Uneducated	39 [15.4]	8 [8.3]	0.10^†^
Below matric	59 [23.1]	18 [18.9]	
Matric & Above	156 [61.5]	70 [72.8]	
			
***Family structure***			
Nuclear	94 [37.0]	64 [66.7]	<0.05^†^
Extended	160 [63.0]	32 [33.3]	
***F/H of obesity***			
Yes	182 [71.7]	40 [41.7]	<0.05^†^
No	72 [28.3]	56 [58.3]	

‡ Mann-Whitney U test

* T-test for independent samples

^† ^Chi-square test of independence

The univariate logistic regression indicated that risk of physical inactivity increased with increasing age and BMI. Married and uneducated subjects were at higher risk for physically inactivity. Individuals from an extended family system and those with a positive family history of obesity were more likely to be inactive. Gender, ethnicity and income were not significantly associated with physical inactivity at the univariate level (Table 3).

**Table 3:** Univariate logistic regression analysis of socio-demographic characteristics associated with physical inactivity *(n = 350)*

Variables	OR (95% CI)	p-value
**Age**	1.36^‡ ^(1.22, 1.52)	0.01
		
**BMI**	1.10 (1.02, 1.16)	0.01
		
**Marital Status**		**<0.05**
Single	1	
Married	3.14 (1.60, 6.15)	<0.05
Widow/er	1.67 (0.35, 7.93)	0.52
		
**Level of Education**		**0.10**
Matric & Above	1	
Uneducated	2.19 (0.97, 4.92)	0.05
Below matric	1.47 (0.81, 2.68)	0.21
		
**Family Structure**		
Nuclear	1	<0.05
Extended	3.40 (2.07, 5.58)	
		
**F/H of Obesity**		
No	1	<0.05
Yes	3.54 (2.17, 5.77)	
		
**Gender**		
Female	1	0.52
Male	1.17 (0.73, 1.87)	
		
**Ethnicity**		**0.22**
Urdu	1	
Other	1.35 (0.84, 2.17)	
		
**Total monthly Income**		
Income	0.996^§^(0.984,1.008)	0.54

^‡ ^OR for every 10 years increase in age

§ OR for every 1000 Rupees increase in income

Hosmer and Lemeshow goodness of fit test indicated that the model fits the data well (χ2 = 3.65, df = 8, p = 0.88).

The final multivariable model (Table 4) included age, BMI, family history of obesity, education, marital status and an interaction between family structure & gender. Income, though not significant, was included in the model as it had a confounding effect on education. The scale of the continuous predictor variables in the model was examined using quartile analysis in logistic regression that indicated a binary scale for age (≤ 33 & > 33 years) and BMI (≤ 33 & > 33 kg/m^2^) respectively. After adjusting for the effect of other variables in the model, subjects older than 33 years were twice more likely to be physically inactive relative to younger subjects. Subjects with BMI > 33 kg/m^2 ^were more likely to be physically inactive relative to those with BMI ≤ 33 kg/m^2^. Subjects with a positive family history of obesity were 3.5 times more at risk of being inactive relative to those without a family history. There was a marginal independent association of being currently married and not having had any formal education with physical inactivity respectively. Males from the nuclear family structure were not significantly different from females of nuclear families with respect to PA. However, females living in extended families were about twice more likely and males living in extended families were six times more likely to be physically inactive as compared to females living in nuclear families.

**Table 4:** Multivariable logistic regression analysis of socio-demographic characteristics associated with physical inactivity among obese attendants *(n = 350)*

Variables	No. of participants [%]	Adj. OR	95% CI
Age*			
≤ 33	91 [26.0]	1	
>33	259 [74.0]	2.01	1.07, 3.78
			
BMI*			
≤ 33	273 [78.0]	1	
> 33	77 [22.0]	2.30	1.12, 4.81
			
**Marital Status**			
Single	40 [11.4]	1	
Married	302 [86.3]	2.07	0.87, 4. 92
Widow/er	8 [2.3]	1.23	0.19, 7.74
			
**Level of Education**			
Matric and above	226 [64.6]	1	
Uneducated	47 [13.4]	2.05	0.82, 5.13
Below matric	77 [22.0]	1.27	0.63, 2.54
			
**F/H of Obesity**			
No	128 [36.6]	1	
Yes	222 [63.4]	3.51	2.05, 6.01
			
**Interaction B/W Family structure & Gender**			
Female in nuclear family	93 [26.6]	1	
Female in extended family	94 [26.9]	2.37	1.16, 4.92
Male in nuclear family	65 [18.6]	1.02	0.49, 2.15
Male in extended family	98 [28.0]	6.15	2.55, 13.27

* Binary categories were made after quartile analysis

Model is adjusted for monthly household income

In Table 5 distribution of responses for various barriers to physical activity among inactive study participants are reported. Among inactive participants more than a quarter admitted that they had information regarding importance of physical activity. Lack of motivation to do PA and not enjoying PA was reported by more than half of obese inactive attendants, whereas more than three-fourth reported that they did not have skills for PA. Only about a quarter of individuals reported presence of any illness that led to physical inactivity. Regarding ***social barriers*** about half of the individuals reported absence of spouse and children's support for PA respectively, while more than half reported absence of other family member's support for PA. Lack of counseling by health care provider, lack of access to places for PA, lack of cost effective facilities, lack of time and lack of safe outdoor environment to some extent were found to be important ***environmental barriers ***among inactive obese individuals.

**Table 5:** Distribution of responses for barriers regarding physical inactivity in inactive obese attendants *(n = 254)*

Barriers	Responses			
	
	Yes	up to some extend	No	N/A
	Numbers (%)	Numbers (%)	Numbers (%)	Numbers (%)
***Personal Barriers***				
Do you have information about the importance of physical activity?	72 (28.3)	126 (49.6)	56 (22.0)	-
Do you have motivation to do physical activity?	91 (35.8)	22 (8.7)	141 (55.5)	-
Do you enjoy physical activity?	103 (40.5)	19 (7.5)	132 (52.0)	-
Do you have skills to do physical activity?	20 (8.3)	18 (7.1)	216 (84.6)	-
Do you feel embarrassed while doing physical activity?	18 (7.1)	3 (1.2)	233 (91.7)	-
Do you have illness/illnesses which interfere with physical activity?	67 (26.4)		187 (73.6)	-
***Social Barriers***				
Do you have spouse's support to do physical activity?	102 (40.2)	9 (3.5)	122 (48.0)	21 (8.3)
Do you have children's support to do physical activity?	88 (34.6)	9 (3.5)	116 (45.7)	41 (16.0)
Do you have other family member's support to do physical activity?	87 (34.3)	9 (3.5)	158 (62.2)	-
***Environmental Barriers***				
Have you been counseled about importance of physical activity by your doctor?	98 (30.0)	101 (34.0)	55 (36.0)	-
Do you have access to places to do physical activity?	64 (25.2)	13 (5.1)	177 (69.7)	-
Do you have cost effective facilities for physical activity?	72 (28.3)	54 (21.3)	128 (50.4)	-
Do you have safe outdoor environment to do physical activity?	120 (47.2)	108 (42.5)	26 (10.3)	-
Do you have time for physical activity?	114 (44.9)	-	140 (55.1)	-

## Discussion

Obesity and physical inactivity are growing problems that are associated with major health problems. To the best of our knowledge, this is the first study to assess the prevalence of physical inactivity in obese individuals, and to determine the barriers to physical activity in inactive obese individuals in Pakistan.

This study has produced some important findings. Firstly, it estimated a high prevalence (72.6%) of physical inactivity in obese subjects. Secondly, this study has recognized the association between physical inactivity and different socio-demographic characteristics. Thirdly, it identified some important personal, social and environmental barriers to PA.

Literature from different parts of the world [19,20] support the high prevalence of physical inactivity found in our study. In a recent study in Saudi Arabia the prevalence of physical inactivity was found to be 40.6%; IPAQ was used to assess physical inactivity, and the study population included both obese and non-obese subjects [20]. In our study only obese individuals were included and hence the prevalence of physical inactivity was found to be higher.

Our study indicates significant and independent association of physical inactivity with age, BMI, family history of obesity, gender and family structure. Our analysis indicated that increasing age exhibits increasing levels of physical inactivity. This has also been indicated in other studies [21,22]. This may be due to increasing domestic as well as professional responsibilities, lack of awareness or poor health as shown by other studies [21,23,24]. Encouragement from physicians and household members may be effective in increasing physical activity among older obese persons. Moreover, our study indicates that higher BMI is associated with physical inactivity, however the causal relationship is difficult to assess in a cross sectional study.

According to our study findings family history of obesity is an important risk factor for physical inactivity that is in agreement with previous studies. Obese family members create obesogenic household environment [25,26]. Family history of obesity on one hand may lead to genetic predisposition for obesity, but on the other hand may reflect behaviors in the family that may lead to sedentary lifestyle [10].

Unlike other studies [22] ethnicity and monthly income were not found to be significantly associated with physical inactivity in our study. The latter may be due to the fact that majority of patients (and attendants) coming to CHC belong to middle-class economic communities. Also absence of association of PA with ethnicity in our study indicates similarity among different ethnic groups in the study population with respect to the PA status. One interesting finding in this study is that both males and females from extended families were more likely to be inactive as compared to females from nuclear families; however males from extended families were more vulnerable. This may partly be due to lack of time for PA secondary to increasing financial responsibilities for males from extended families, however females with large families have more domestic responsibilities as well. In IPAQ domain of domestic activities is extensively explored. In our population, with females being involved more in household activities, IPAQ may lead to appropriate estimation of PA level in females. This finding identifies the need of validation of IPAQ in our setting.

Results from our study highlight considerable personal and environmental barriers to PA among obese individuals. In agreement with reports from previous studies [18-20,27], lack of motivation and lack of time were found to be important barriers. In addition lack of accessibility to places for PA, lack of cost effective facilities, lack of safe outdoor environment, inadequate counseling by physicians were also found to be important barriers. Results of this study and few others indicate that only a small number of obese adults received advice by a health care professional about physical activity [28-30].

An important role of physicians and other health care professionals is to assist individuals to adopt healthy lifestyle habits related to diet and exercise. Physician understanding and discussion of potential barriers and development of an individualized physical activity program can lead to a greater compliance. It has been shown that discussing the benefits of physical activity, barriers to physical activity, patient preferences & practices need not take more than three to five minutes of a physician's time [31].

The major strengths of our study are; this is the first study so far to assess the prevalence of physical inactivity in obese individuals and to identify the barriers to PA in accordance with individual perceptions, recall bias regarding physical activity being minimized as IPAQ focuses on previous week's physical activities. Moreover, IPAQ has a detailed domestic PA domain that identified domestic work done by women who usually do not work outside home in our cultural setting. We used the latest recommended BMI cut-offs for Asian populations. There was a good response rate of about 95%. Some practical recommendations based on study results have also been suggested. However there are few *limitations *such as the study was cross-sectional in design that prevents determining causation, our study participants may not be representative of the general population which may limit its generalizability. The tool used to measure physical activity (IPAQ) is not validated in Pakistan in local language; nonetheless it has been validated in many countries. Moreover, another limitation is that reference [18] studies barriers to physical activity among young women only. However, the questionnaire that was used in reference [18] was based on review of literature investigating barriers to physical activity among general adult population. For the present study the barriers identified from [18] were modified to adapt to the local culture and to extend to individuals aged 18 years and above.

## Conclusion and recommendation

Sedentary life style is growing with urbanization and modernization. Considering the public health implications of physical inactivity it is essential to promote PA, however that should be in the context of an individual's health and perception. It is vitally essential for primary care provider to help individuals to establish realistic expectations and select their own preferences.

Our findings highlight considerable individual, social and environmental barriers to PA among obese subjects that need to be addressed during counseling sessions regarding PA.

## List of abbreviations

AKUH: Aga Khan University Hospital; BMI: Body Mass Index; CHC: Community Health Center; CI: Confidence Interval; ERC: Ethical Review Committee; MET: Metabolic Equivalent; NCD: Non-Communicable Diseases; NHSP: National Health Survey of Pakistan; OR: Odds Ratio; PA: Physical Activity; Rs: Rupees (Pakistani Currency, 1US$ = 60Rs.); SD: Standard Deviation; SPSS: Statistical Package for Social Sciences

## Competing interests

The authors declare that they have no competing interests.

## Authors' contributions

NS: conception and design of the study, analysis and interpretation of the data, drafting the manuscript. SM: Assisted during protocol development stage, in analysis and interpretation of the data, critically revising the article. AKK: assisted in study design, critically revising the article. All contributors approved the final version of the manuscript.
